# A Woman with Recent Jaundice and Pruritus

**Published:** 2010-03-01

**Authors:** Bita Behnava, Seyed Moayed Alavian

**Affiliations:** 1 Baqiyatallah Research Center for Gastroenterology and Liver Disease,Baqiyatallah University of Medical Sciences, Tehran, I.R.Iran

**Keywords:** Jaundice, Pruritus, Cholestasis

## Abstract

A middle-aged woman suffering from jaundice and pruritus that had begun a month previously was presented to a physician.At the first assessment, laboratory findings had revealed a cholestatic pattern and serologic tests for hepatitis B virus (HBV), hepatitis C virus (HCV) and hepatitis A virus (HAV) were negative. Normal findings of abdominal computed tomography (CT) scan and endoscopic retrograde cholangiopancreatography (ERCP) ruled out extrahepatic causes of cholestasis. A liver biopsy was done and showed intrahepatic cholestasis without destruction of the bile ducts or granuloma.We assessed the intrahepatic causes of cholestasis. Finally the diagnosis was confirmed by means of a simple test.

## Case Presentation

A 41-old-woman suffering from jaundice, pruritus and fatigue which had begun 6 days previously, presented herself to her general practitioner (GP). She did not have any fever, nausea, diarrhea, anorexia, abdominal pain, rash, respiratory symptoms or arthralgia. She had lost five kilograms of weight over the preceding two months. At the time she visited her GP, her weight was 49 kg and her height 159 cm. The patient did not have any history of blood transfusions or alcohol use. She had traveled to a rural area in northern Iran two months previously. Her family history was negative for liver disease. For the preceding three months she had been taking chlordiazepoxide without a prescription. For the past five months, she had been taking a weekly dose of three glasses of orange flower water as a herbal remedy.

Laboratory analysis showed a white blood cell (WBC) count of 9100/µL of which 70% was neutrophil, 23% lymphocyte, 6% eosinophil and 1% monocyte. The alanine aminotransferase (ALT) level was 69 IU/ml (normal range 10 to 40), the aspartate aminotransferase (AST) level 61 IU/ml (normal range 15 to 40), the alkaline phosphatase (ALP) level 525 IU/ml (normal range 45 to 150), the gamma-glutamyl transferase (GGT) level 410 IU/ml (normal range 10 to 80), the total bilirubin level 10 mg/dl(normal range:0.3 to 1.3), the direct bilirubin level 8.7(normal range:0.1 to 0.3), amylase level 70 IU/ml (normal range), albumin level 4 g/l, gamma glubolin 1.6 g/l, prothrombin time 12 second, INR 1.1 and first hour ESR 20. Seromic IgG and IgM levels were normal. Serologic tests for hepatitis B virus (HBV), hepatitis C virus (HCV) and hepatitis A virus (HAV), human immunodeficiency virus (HIV) and also anti mitocondrial antibodies, anti smooth muscle antibodies and anti liver kidney microsomal antibodies were negative. Anti nuclear antibodies wereat a very low positive level. Abdominal sonographic findings were normal and there was no dilation or obstruction of the biliary ducts.

Ten days later, the patient returred to the clinic with nausea, vomiting and worsening jaundice and pruritus. She was referred to a hospital for further evaluation. On admission, the patient was agitated and suffering from severe jaundice, nausea and pruritus. Her temperature was 37.6 C, her pulse 115 beats per minute, her blood pressure 130/80 mmHg and her respiratory rate 18 per minute. The patient had mild tenderness on right upper quadrant (RUQ) and her skin was moist; the remainder of the examination was normal. The results of laboratory tests revealed an ALT level 70 IU/mL, an AST level 61 IU/mL, an ALP level 590 IU/mL,GGT 389 IU/mL, a total bilirubin level 28 mg/dl, bilirubin direct 23.6mg/dl, angiotensine converting enzyme 65 IU/mL (normal range). A chest x ray and computed tomography (CT) scan of the abdoman and pelvis showed no abnormalities. Serologic tests for Epstein-Barr virus (EBV), cytomegalovirus (CMV), hepatitis E virus (HEV), brucella and fasciola hepatica were negative. Endoscopic retrograde cholangiopancreatography (ERCP) did not show any abnormality. A liver biopsy was done and revealed bile plugs in the canaliculi, pigment accumulation in the cytoplasm of some of the hepatocytes, an increase in Kupffer cells with pigment deposition in the perisinusoidal space, and a regular appearance of bile ductules, compatible with intrahepatic cholestasis (canalicular cholestasis).

In view of her tachycardia, moist skin, agitation, hand tremor and unknown cholestasis, thyroid function tests were obtained. Serum thyroid hormone levels were: free thyroxine (FT4) 6.17 ng/dl (normal range: 0.8-1.9); free triiodothyronine (FT3) 10.2 ng/dl (normal range: 1.8-4.2); thyroid-stimulating hormone (TSH) less than 0.01 mU/ml (normal range: 0.4-4); and anti-thyroid peroxidase 399(normal range: 0-35). Thyroid scintigraphy (3 mCi Tc 99 m pertechnetate IV) indicated a diffuse high-level technetium uptake.

She was diagnosed with Graves' disease. Propylthiouracil (PTU) was started at a daily dose of 300 mg. After five days of therapy, pruritus and jaundice had worsened (total bilirubin had increased to 31mg/dL and her ALP to 670 IU/ml) so PTU was stopped and radioactive iodine (I131) was initiated for thyroid ablation. Nausea and pruritus were eliminated after one week and jaundice improved when thyroid hormone levels became normal after five weeks.

## Discussion

The initial presentation of the patient suggested possible diagnoses of viral hepatitis, alcoholic hepatitis, hepatotoxic drugs or remedies, autoimmune hepatitis or cholestatic syndrome. Serologic tests for hepatotropic viruses were negative. On the basis of history, alcoholic hepatitis could be ruled out. Chlordizepoxide has no hepatotoxic side effects and also orange flower water was unlikely to be a cause for her disease. The levels of gamma globulin, Immunoglobulin G (IgG) and Immunoglobulin M (IgM) were normal and also autoantibodies were negative; thus a diagnosis of autoimmune hepattitis was unlikely. The pattern of elevated liver enzymes, alkaline phosphatase and bilirubin was consistent with a cholestasis possibly due to intra- and extrahepatic causes. Sonography of her right upper quadrant did not show any extrahepatic obstruction. Despite the high degree of sensitivity and specficity of this imaging technique, some causes of obstruction may be missed. As the next step for evaluation of probable extahepatic cholestasis, a CT scan and an ERCP were done. We discuss the causes of extra-and intrahepatic cholestasis in this case.

### Extra-hepatic causes

Choledocholithiasis: In a 41-year-old woman with a cholestatic presentation, choledocholithiasis as the most common cause of extrahepatic cholestasis should be considered. It can cause a range of symptoms from mild right upper quadrant pain with only minimal elevations of alkaline phosphatase, to jaundice and fever due to ascending cholangitis. However, gall bladder sonography and ERCP did not show any stones.

Malignancies: Cholestatic jandice and significant weight loss (nearly 10% of total weight) in the present case would suggest the probability of malignancies. Tumors of the pancreas, gallbladder, ampullary, cholangiocarcinoma and portal adenopathy can cause extrahepatic cholestasis. However, in this case the normal findings of abdominal sonography, a CT scan and an ERCP ruled out these causes.

Fasciola hepatica: The patient had travelled to a village in northern Iran and eaten fresh vegetables two months before the onset of her disease and also had had a mild eosinophilia in the peripheral blood smear. Cholestatic hepatitis and eosiniphilia in a patient with a history of travelling to an endemic region for fasciola hepatica should be suspected in forming this diagnosis. Fascioliasis was divided into two phases: in the liver phase, the larvae migrate through the liver tissue, there is pain in right upper quadrant, and fever and sometimes jaundice may occur. In the biliary phase, the adult flukes may obstruct the common duct and is associated with cholestatic jaundice [1][2]. Eosinophilia is a variable finding in this phase of the disease. However, serologic testing for fasciola hepatica was negative and ultrasound and ERCP findings were normal.

Pancreatitis: One of the complications of chronic pancreatitis is bile duct stricture and cholestatic jaundice [3]. This is most commonly seen in patients with a long history of epigastric pain and pancreatic insufficiency, but she did not have such a history and also her serum amylase level and the ERCP findings were normal. In view of the normal CT scan and ERCP findings, extrahepatic causes of cholestasis were unlikely. However the intrahepatic causes remain as a major diagnosis. A liver biopsy was done to determine the etiology of her disease and was consistent with an acute canalicular cholestasis without ductopenia.

### Intrahepatic causes

Viral infections: Many of the virus infections that typically cause a hepatocellular pattern of injury can also present as a cholestatic feature such as HAV, HEV, EBV, CMV [4][5][6][7][8]. Hepatitis A can be followed by a prolonged cholestasis that may last 8 to 12 weeks and is usually characterized by severe jaundice(with a total bilirubin of more than 10mg/dL, a marked elevation of ALP and a mild elevation of aminotrasferases), pruritus, fever, weight loss, diarrhea, and malaise [9]. Also, a few cases of cholestasis have been reported in patients with HEV infection [10]. One of the clinical manifestations of CMV and EBV infections is hepatitis, which can occur in patients with febrile syndrome. In some of these patients,a cholestatic feature may predominate [5][6], although the patient did not have fever and her serologic tests for all of these infections were negative.A cholestatic feature in HBV and HCV infections (fibrosing cholestatic hepatitis) may present after solid organ transplantation [11][12][13].

Drugs and herbal remedies: Several drugs can cause intrahepatic cholestasis, including amitriptyline, ampicillin, amoxicillin-clavulanate, carbamazepine, chlorpromazine, cyproheptadine, erythromycin estolate, haloperidol, imipramine, prochlorperazine, phenytoin, trimethoprim-sulfamethoxazole, tetracycline and atorvastatin [14][15][16][17][18]. The patient had used chlordiazepoxide for anxiety, but it does not seem that this drug could cause cholestasis. Herbal remedies should be considered as a cause of unknown cholestatic hepatitis. Several case reports have shown the association between some of the natural remedies such as senna and Cascara sagrada and cholestatic hepatitis [19]. The patient had used orange flower water for five months, but it does not appear to cause cholestatic hepatitis. PTU is listed among the drugs that can cause intrahepatic cholestasis [20][21][22]. After using PTU as an antithyroid therapy in this patient, her jaundice was aggravated.

Primary sclerosing cholangitis (PSC): PSC is characterized by the destruction and fibrosis of the intrahepatic and/or extrahepatic bile ducts. The majority of patients with this condition are male and have underlying ulcerative colitis [23][24]. Fatigue and pruritus are common features at presentation, but persistent jaundice usually heralds advanced disease. Hypergammaglobulinemia, increased serum IgM levels and atypical perinuclear antineutrophil cytoplasmic antibodies (P-ANCA) may be seen in these patients. ERCP findings include multifocal stricturing and dilation of intrahepatic and/or extrahepatic bile ducts. However in this case, ERCP findings were normal.

Primary biliary cirrhosis:  Primary biliary cirrhosis (PBC) is characterized by a progressive autoimmune destruction of small intralobular bile ducts and finally results in cirrhosis and liver failure [25]. It typically presents in middle-aged women. Fatigue and pruritus are the most common presenting symptoms of PBC. Like PSC, jaundice is most often a late symptom that reflects advanced liver disease [25]. It should be noted that in prolonged intrahepatic cholestasis with jaundice,the presence of signs of advanced liver disease suggests PBC, PSC or overlapping syndromes. Hyperpigmentation, hepatosplenomegaly and osteopenia can be seen in these patients. The finding of antimitochondrial antibodies (AMA) seropositivity is highly sensitive (95% to 98%) as a diagnostic test. Nevertheless, the diagnosis of PBC should be confirmed by liver biopsy. The early stages of PBC are characterized by portal hepatitis, granulomas and bile duct lesions, but in progressive stages fibrosis spanning portal tracts and ductopenia are dominated [26]. The cholestatic feature in the present case was not followed by any signs of advanced liver disease. Also her AMA test was negative and her liver histological features showed an intrahepatic cholestasis without any bile ductule lesions or granulomas which is not compatible with primary biliary cirrhosis.

Autoimmune cholangitis: Autoimmune cholangitis is typically found in groups of patients who have histologic features suggesting PBC but are often seronegative for AMA, and generally have circulating antinuclear antibodies (ANA) and/or smooth muscle antibodies (SMA) [27]. We considered the probability of autoimmune cholangiopathy in a middle aged woman with unknown cholestasis, autoimmune hyperthyroidism who tested positive for ANA, but the patient had a very low level of anti nuclear antibodies in her serum and normal levels of IgG and IgM, and also the histologic findings were not consistent with PBC or autoimmune choangiopathy.

Sarcoidosis: Sarcoidosis is a multisystem granulomatous disorder and is characterized by noncaseating granulomas in involved organs. Hepatic involvement is most often asymptomatic (only elevation of alkaline phosphatase and gamma glutamyl transpeptidase) but rarely cholestasis and cirrhosis can occur [28][29]. In the present case, the absence of clinical signs of the disease in other organs, a normal chest x ray, a normal ACE level and histologic findings (intrahepatic cholestasis without granulomas) would exclude sarcoidosis.

Stauffer's syndrome: This paraneoplastic syndrome is a reversible intrahepatic cholestasis, in the absence of liver metastases. Stauffer's syndrome is most commonly associated with renal cell carcinoma and less common with malignant lymphoproliferative disease and gynecologic malignancies [30]. In this case, however, the CT scan of abdomen and pelvis was normal.

### Hyperthyroidism

At first visit the physician did not consider the signs of hyperthyroidism, but when the patient was hospitalized, in view of the patient's tachycardia, moist skin, agitation, hand tremor and unknown intrahepatic cholestasis, thyroid function tests were ordered. Hyperthyroidism with high level anti-thyroid peroxidase and diffusely uptake on thyroid scan confirmed the diagnosis of Graves' disease. It seems that her intrahepatic cholestatic jaundice had developed secondarily to autoimmune hyperthyroidism in the absence of congestive heart failure and was worsened by PTU. It was confirmed that when euthyroidism occurred, her jaundice was cured. Thyroid disorders can cause abnormalities in aminotransferases and alkaline phosphatase levels. Hyperthyroidism can rarely be associated with intrahepatic cholestasis [31][32][33][34]. The underlying mechanisms were unclear, but some hypotheses have explained the probable etiology of liver enzyme abnormalities and cholestasis in hyperthyroidism patients. One of these hypotheses, has suggested that in the absence of heart failure, hypoxia due to a hypermetabolic state can damage centrilobular zones and result to canalicular cholestasis [32][33]. In one study on rats, liver dysfuntion in hyperthyroidism has been attributed to mitochondrial dysfunction `[35]. Antithyroid drugs including methimazole or PTU can also induce or contribute to the cholestatic jaundice [32][36][37]. It should be noted that cholestatic jaundice is a rare complication of hyperthyroidism and if it does occur the probability of heart failure, concomitant with autoimmune hepatitis or other underlying liver disease and drug hepatotoxicity should be considered.
